# Successful full-term pregnancy after preterm event in a liver transplant patient: a case report 

**DOI:** 10.1186/s13256-023-04189-1

**Published:** 2023-12-10

**Authors:** Ramona Galsone, Sandra Vītiņa, Maira Jansone, Aiga Staka, Reza Mohammadian

**Affiliations:** 1 Women and child health clinic, Pauls Stradins Clinical University Hospital, University of Latvia, Pilsonu street 13, Riga, 1002 Latvia; 2 Gastroenterology, Hepatology and Nutrition Therapy Center, Pauls Stradins Clinical University Hospital, Pilsonu street 13, Riga, 1002 Latvia; 3 Radiology Department, Riga East University Clinical Hospital, Stradins University, Hippocrates Street 2, Riga, 1038 Latvia

**Keywords:** Liver transplantation, Pregnancy, Risks, Benefits, Personalized approach

## Abstract

**Background:**

Pregnancy after liver transplantation poses a significant challenge to both the patient and the transplant team.

**Case presentation:**

We present the case of a 19-year-old European patient who underwent liver transplantation 5 years previously owing to autoimmune hepatitis. Poor compliance with immunosuppressive therapy and missed follow-up visits during the patient’s first pregnancy likely contributed to her liver function deterioration, hospitalization, and failed pregnancy. Owing to the patient’s complex medical history, combined immunosuppressive treatment, and risks to the fetus, her second pregnancy was high risk. However, close outpatient monitoring and adherence to treatment led to a successful, uneventful, full-term pregnancy and healthy delivery.

**Conclusion:**

Liver transplant recipients who desire to become pregnant require careful planning and management to ensure optimal outcomes for both the mother and the fetus. A personalized strategy is necessary to balance the potential benefits of childbirth with the risks involved in pregnancy after liver transplantation.

## Introduction

Chronic liver disease in women can have a significant impact on their reproductive health, with a very low chance of conceiving and carrying a pregnancy. The principal cause of this is often due to the disruption of the hypothalamic–pituitary–ovarian axis that regulates the menstrual cycle and hormonal balance in the body. Severe chronic liver disease can lead to amenorrhea, which further exacerbates the situation. This condition affects up to 50% of women of childbearing age and can lead to hormonal imbalances, such as hypogonadotrophic hypogonadism and elevated levels of estrogens, that further decrease the chance of successful conception. As a result, women with end-stage liver disease often face difficulties in conceiving and carrying a pregnancy to term [1–4].

Liver transplantation presents a hopeful opportunity for women who have experienced difficulty conceiving owing to chronic liver disease. Following the transplantation procedure, female fertility is often restored, with studies showing that fertility can return as early as 6 weeks post-surgery. Almost 90% of female recipients also report experiencing regular menstrual periods within a year of the transplant, indicating a positive impact on their reproductive health. This provides a potential solution for women who previously faced challenges in becoming a mother owing to chronic liver disease [5, 6].

Liver transplantation has a history of significant milestones, with the first successful liver transplant being performed in 1963 by American surgeon Thomas Starzl. A groundbreaking moment occurred in 1978 when the first reported successful pregnancy in a liver transplant recipient was documented, highlighting the potential for women to experience successful pregnancies after liver transplantation [7].

Liver transplant numbers have consistently increased among women of reproductive age, as reported by the European Liver Transplant Registry. It has been estimated that about 75% of female liver transplant recipients have childbearing potential [5]. As a result, there is a growing number of cases of successful pregnancies following liver transplantation. However, uncertainties persist regarding preconception counseling and the best approach for managing pregnancy in these individuals [8, 9].

Pregnancy itself is a multifaceted process. There are additional complexities and uncertainties to consider for women who have undergone solid organ transplantation. These include potential risks to the mother’s long-term health and ability to parent, the possibility of allograft dysfunction or even graft loss related to the pregnancy itself, and the potential changes of drug metabolism during pregnancy that could increase the risk of transplant rejection. Furthermore, there are potential risks to the fetus or neonate, including teratogenic effects associated with immunosuppressive and other medications. These risks may not be limited to gross structural defects but also could manifest as subtle developmental changes that may only become apparent later in life [10, 11].

This article presents a case report detailing the experience of a female liver transplant recipient who underwent both an unplanned, unsuccessful pregnancy and a subsequent successful pregnancy, ultimately resulting in favorable maternal and fetal outcomes. The findings of this case study offer valuable insights into the clinical implications and potential challenges of pregnancy in the context of liver transplantation.

## Case presentation

### First pregnancy

A 19-year-old European patient was transferred to our hospital at 27 weeks 6 days of her first pregnancy owing to her health condition deterioration. She was married, worked in a safe environment, and denied any harmful habits. Contraception was through the use of condoms. There were no chronic or oncological diseases in her family history. She was transferred from a regional hospital to a tertiary hospital owing to complaints of moderate, dull pain in her abdomen, mainly in the right upper quadrant, and progressive jaundice. However, fetal movements were felt to be good. It was noted that she had been using a lower dose of immunosuppressive therapy than had been prescribed and recommended by gastroenterologists.

According to the patient’s medical history, she received a domino-type liver transplant of the right lobe due to autoimmune, hepatitis-induced cirrhosis 5 years previously. Unfortunately, she was not adherent to immunosuppressive therapy, which caused a relapse of autoimmune hepatitis in the graft with a subsequent hospitalization with high cytolysis and hyperbilirubinemia (221 μmol/L) 1 year previously. Methylprednisolone pulse therapy (1000 mg intravenously for 3 consecutive days) was prescribed, which had a positive therapeutic effect. The patient was strongly advised to follow immunosuppressive medication, and possible consequences of nonadherence were explained. During the following 10 months, her liver function tests were almost within the normal range. However, after 11 months, the patient experienced unexpected pregnancy and decided to continue the pregnancy. At week 12, her blood, liver function tests, and tacrolimus trough level were within normal range when reviewed by a gastroenterologist during an outpatient visit. The patient was informed of the risks of immunosuppressive therapy nonadherence as well as explained that the pregnancy was considered high risk. She was recommended to strictly follow the immunosuppressive therapy regimen consisting of tacrolimus, azathioprine (if regular tacrolimus trough level), and liver function test control. The patient had a scheduled follow-up visit with blood analysis 1 month later. Unfortunately, she did not attend subsequent outpatient control visits.

Upon admission to the hospital, the patient took tacrolimus, azathioprine, ursodeoxycholic acid, and vitamin D with calcium supplements irregularly.

On physical examination, the patient appeared to be conscious, active, and adequately responsive. The skin and mucous membranes were slightly icteric. She was hemodynamically stable with normal respiration function. The abdomen was soft to palpation but diffusely sensitive. There were no signs of peritoneal irritation, and urination was reported as unhindered but slightly darker in color.

The fetal heart rate was measured at 146 beats per minute and rhythmic, with healthy amniotic fluid and the uterus normotonic.

On the third day, fetal ultrasound was performed that showed the fetus’ size corresponded to the 28th week 2 days, with the head recumbent. The fetal heart rate was 143 beats per minute and rhythmic, and amniotic fluid was normal. The placenta was placed high on the anterior wall, and the cervix was closed and 43 mm long.

**Fig. 1:**
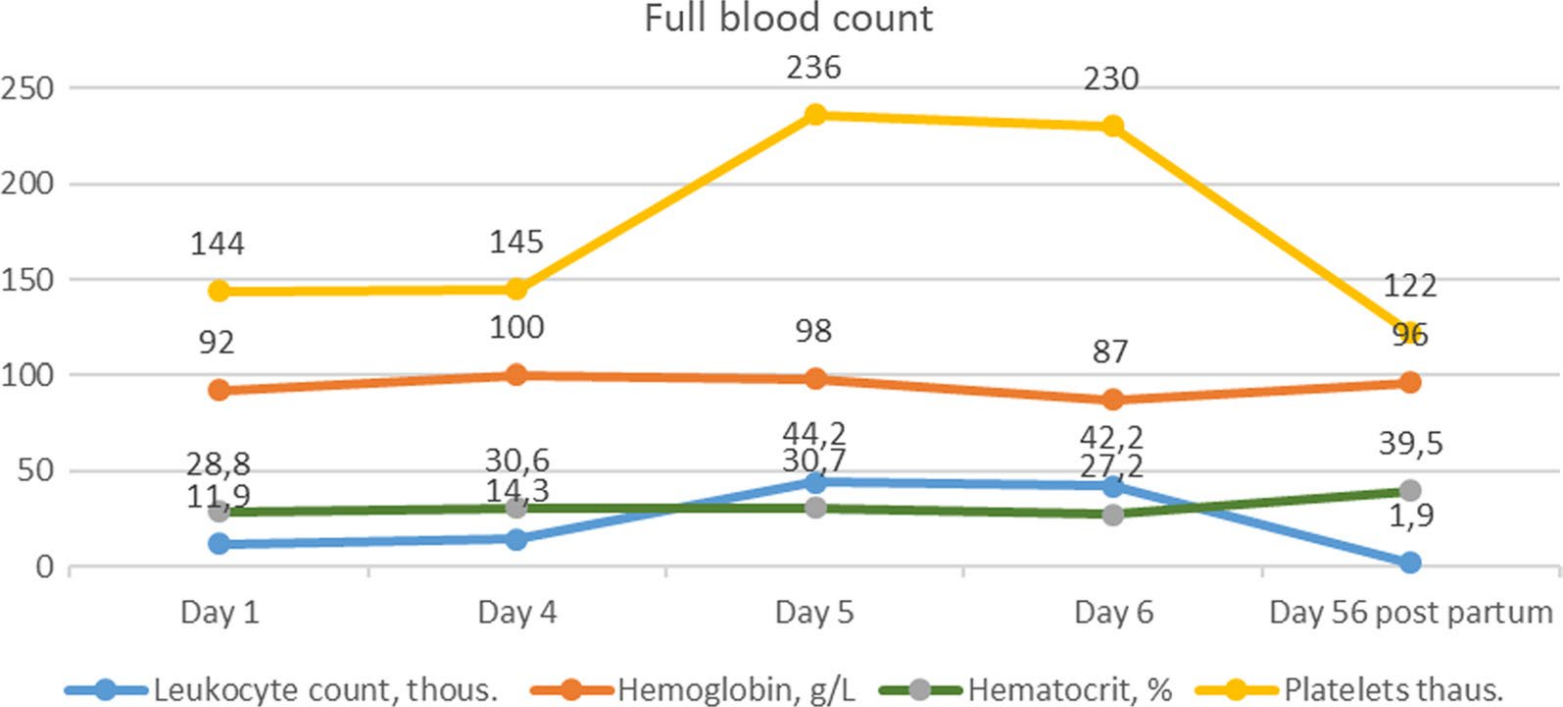
Full blood count dynamic changes in first pregnancy

**Fig. 2:**
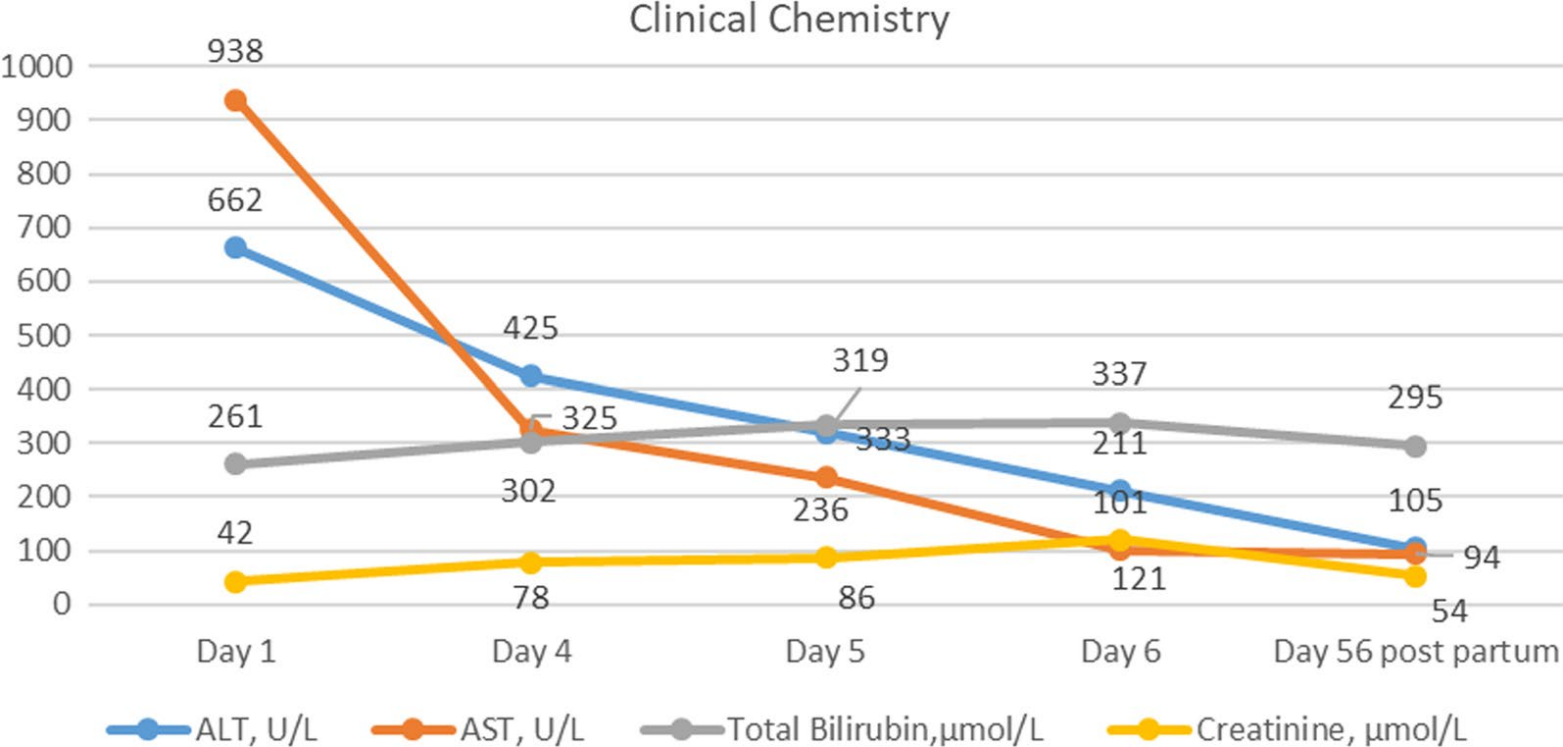
Clinical chemistry dynamic changes in first pregnancy

Peroral therapy with methylprednisolonum of 32 mg daily was commenced. On the fourth day of hospitalization, additional 500 mg of methylprednisolone was administered intravenously for three consecutive owing to increasing cytolysis. On the fifth day of admission, the patient experienced diffuse abdominal pain, which was treated with antianalgesic therapy. Abdominal ultrasound revealed signs of portal hypertension, including splenomegaly, portal vein dilatation, small ascites, and mild (grade 1) bilateral urostasis associated with advanced pregnancy. However, the patient’s condition worsened and hepatic encephalopathy progressed—the patient became sluggish and drowsy. On the sixth day, due to hepatic encephalopathy progression (Figs. 1,2), the patient was transferred to the intensive care unit (ICU).

On the morning of the sixth day, a multidisciplinary *concilium* including an obstetrician, gastroenterologist, infectologist, intensive care unit physician, and anesthesiologist concluded that urgent caesarean section was necessary owing to the rapid deterioration of the patient’s liver failure, including hyperbilirubinemia, coagulopathy (Table 1), and encephalopathy. The operation was considered to be high risk owing to the patient’s general condition and required prepreparation with blood components. At 27/28 weeks, the fetus was viable, but further development could not be predicted owing to the mother’s high hyperbilirubinemia and potential fetal brain damage.

**Table 1:** Laboratory test results during the first pregnancy

Parameter	Day 1	Day 4	Day 5	Day 6
Leukocyte count × 10^9^/L	11.9	14.3	44.2	42.2
Erythrocyte count × 10^12^/L	3.96	4.25	4.29	3.8
Hemoglobin, g/L	92	100	98	87
Hematocrit, %	28.8	30.6	30.7	27.2
Platelets × 10^9^/L	144	145	236	230
Absolute neutrophil count × 10^9^/L	8.9	13	40.1	37.9
Absolute lymphocyte count × 10^9^/L	1.6	1.0	2.1	2.1
Absolute eosinophil count × 10^9^/L	0.2	0.0	0.0	0.3
ALT, U/L	662	425	319	211
AST, U/L	938	325	236	101
Gamma-glutamyl transpeptidase, U/L	11	12	15	22
Total bilirubin, µmol/L	261	302	333	337
Direct bilirubin, µmol/L	211	256	274	280
Indirect bilirubin, µmol/L	50	54	59	57
Total protein, g/L	51	—	—	46
Albumin, g/L	30	—	—	26
Urea, mmol/L	2.6	—	—	9.7
Creatinine, µmol/L	42	78	86	121
APTT, sec	41.2	—	47.2	41.1
Prothrombin index, %	42.3	—	37.2	38.9
INR	1.58	—	18	—
Fibrinogens, g/L	2.03	—	1.77	1.70
Antitrombin III, %	43.3	—	1.47	0.99

ALT: Alanine Aminotransferase, AST: Aspartate Aminotransferase, APTT: Activated Partial Thromboplastin Time, INR: International Normalized Ratio

**Fig. 3:**
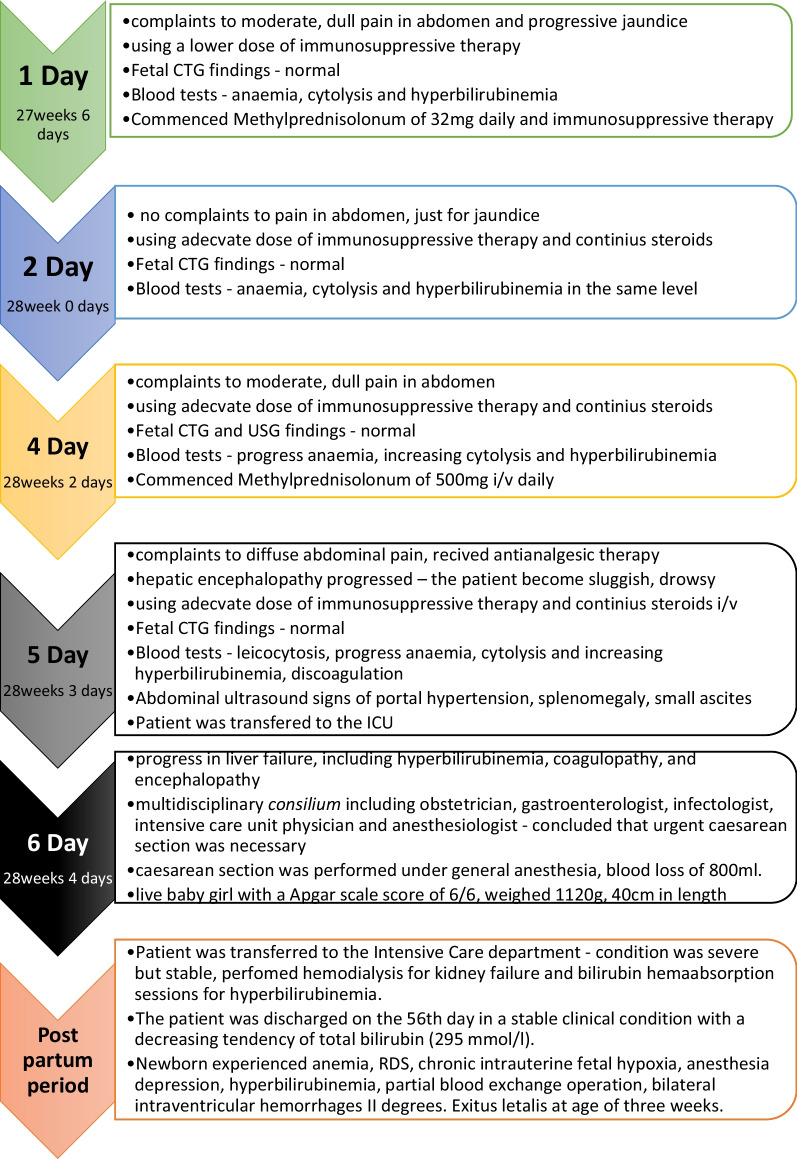
Events in first pregnancy

On the same day, a lower laparotomy caesarean section was performed under general anesthesia, resulting in the birth of a live baby girl with Apgar scale score of 6/6. The newborn weighed 1120 g and measured 40 cm in length, 23 cm in chest circumference, and 27 cm in head circumference. The placenta separated spontaneously, weighing 405 g, with blood loss of 800 ml. After the operation, the patient was transferred to the intensive care department (Fig. 3).

The newborn experienced irregular breathing after birth and received respiratory support, requiring both positive end-expiratory pressure (PEEP) and assisted ventilation. Owing to planned surfactant administration, the child was intubated and transferred to the ICU. Enteral feeding through a gastric tube with an adaptive mixture was started on the second day of birth, and the child exhibited adequate diuresis. A Passy Muir valve (PMV), phototherapy, and a partial blood transfusion exchange operation (100 ml/kg filtered, absorbed erythrocyte mass with plasma transfusion to exchange 100 ml/kg circulating newborn blood, performed in newborns with hemolytic disease to reduce hiperbilirubinemia, antibodies of erythrocytes, and sensitized erythrocytes) were administered. On the third day, sedation with fentanyl 2 mcg/kg/hour was given with hemodynamics and adequate diuresis. However, fluctuating oxygen saturation and a heart murmur were detected, along with a large patent ductus arteriosus (PDA) in echocardiography.

The child also experienced anemia. Ultimately, the child was diagnosed with respiratory distress syndrome (RDS), chronic intrauterine fetal hypoxia, anesthesia depression, hyperbilirubinemia, bilateral intraventricular grade II hemorrhages, and anemia on the eighth day. The child was transferred to a children’s hospital, but sadly passed away at the age of 3 weeks.

Postpartum period

During the postpartum period, the patient’s condition was severe but stable after undergoing hemodialysis for kidney failure and two bilirubin hemoadsorption sessions for hyperbilirubinemia in the cardioresuscitation unit. On the sixth day after the cesarean section, the patient was transferred to the gastroenterology department. Immunosuppressive therapy with tacrolimus, azathioprine, and methylprednisolonum 32 mg/day was continued. On the eighth day, a liver biopsy was performed to determine the morphological status of the liver, revealing liver cirrhosis with the formation of pseudolobules and cirrhotic transformation, relapse of autoimmune hepatitis, and no data on transplant rejection. Although the patient’s condition was clinically stable, hyperbilirubinemia continued to progress (424 µmol/l on day 11) and discoagulation remained increased (INR 1.5). After *concilium* decision on the 12th day, methylprednisolone pulse therapy was readministered for 3 days. On the 19th day, due to the poor prognosis of the patient’s disease, the multidisciplinary *concilium* decided to include the patient on the active list for liver transplantation. The patient’s hyperbilirubinemia continued to progress (597 µmol/l on the 25th day), leading to repeated bilirubin hemaabsorption sessions on the 26th, 27th, and 28th days. Viral hepatitis A, B, C, and E were ruled out for the patient.

On the 20th day, the patient’s blood sample showed a positive result for cytomegalovirus DNA (quantitatively 1644 copies/ml). On the 26th day, the viral load increased to 2880 copies/ml. The patient was treated with valganciclovir 900 mg/day orally, based on creatinine clearance. The viral load was negative on the 46th day. The patient also underwent consultation with a neurologist, psychiatrist, and psychologist. The patient was discharged on the 56th day in a stable clinical condition with a decreasing tendency of total bilirubin (295 mmol/l) and INR of 1.18. She continued her care on outpatient basis with the supervision of a family doctor, gastroenterologist, and gynecologist. It was emphasized to the patient that she needed to avoid pregnancy for the next 2–3 years and be adherent to medical therapy.

### Second pregnancy

The patient’s second pregnancy occurred 1 year after her first one. At 14 weeks into the pregnancy, the patient was informed of possible complications by a multidisciplinary *concilium* consisting of a gastroenterologist, infectious disease specialist, gynecologist, and psychotherapist. Despite their recommendation to terminate the pregnancy, the patient opted to continue. Later on, she was admitted to the gastroenterology department of a tertiary hospital for further monitoring and examination.

Tables 2 and 3 present the laboratory tests, ultrasound findings, and medications administered during the second pregnancy at various stages of gestation. During her hospitalization, she received methylprednisolone therapy and continued to take azathioprine, tacrolimus, and ursodeoxycholic acid. The patient had an elective caesarean section at 37 weeks owing to a scar on uterus from a previous caesarean section and patient preference. RDS prophylaxis was administered before the procedure, and the baby girl was born weighing 2860 g and measuring 50 cm with 7/8 points on the Apgar scale score. Blood loss during childbirth was 1170 ml, and the patient required two erythrocyte mass transfusions for anemia in the postpartum period. On the fourth day after the caesarean section, the patient and child were discharged in a generally satisfactory condition and referred to outpatient care with a family doctor, gynecologist, and gastroenterologist.

**Table 2:** Laboratory test results and medications used during second pregnancy at different weeks of gestation

Weeks of Pregnancy	14	16	21	23	25	31	33	35	37
Complaints	None	None	None	None	None	None	None	None	None
Physical findings	None	None	None	None	None	None	None	None	None
Leukocytes × 10^9^/L	7.4	10.9	7.1	6.7	10.7	8.14	10.1	8.14	9.9
Erythrocytes × 10^12^/L	3.75	3.5	3.6	3.6	3.6	3.3	3.5	3.5	3.5
HGB, g/L	97	94	102	106	105	96	102	102	96
Platelets × 10^9^/L	99	108	113	115	119	127	132	—	—
INR	1.02	1.06	1.03	1.08	1.06	1.03	1.08	1.03	1.03
Prothrombin index, %	95.8	85.5	91.7	82.9	81.6	87.4	98.1	—	—
Fibrinogen, g/L	3.01	2.95	2.62	2.75	2.20	—	—	—	—
AST, U/L	209	29	24	22	21	14	50	50	50
ALT, U/L	164	24	15	11	11	12	2.5	3.5	3.5
Total bilirubin, µmol/L	16	9	9.56	8.18	6.29	8	3.2	3.5	3.5
Alkaline phosphatase, U/L	157	74	72	107	106	122	500	500	500
GGT, U/L	90	72	35	17	17	19	800	800	800
Creatinine, µmol/L	39	35	4.4	2.4	3.9	39	1	2	2.5
Tacrolimus concentration, ng/ml	7.9	8	10.3	10.2	10.2	15	30	13	15
Medications used									
Methylprednisalone (mg)	32	24	12	8	8	8	8	8	8
Azathioprine (mg)	50	50	50	50	50	50			
Tacrolimus (mg)	2	2	2	2.5	3	3	3.5	3.5	3.5
Ursodeoxycholic acid (mg)	500	500	500	500	500	500	500	500	500

AST: Aspartate Aminotransferase, ALT: Alanine Aminotransferase, GGT: Gamma-glutamyl transpeptidase

**Table 3:** Ultrasound results for fetal development and other parameters during pregnancy at weeks 20, 31, and 35

Test results for ultrasound	Week 20 of pregnancy	Week 31 of pregnancy	Week 35 of pregnancy
Fetal parameters	Corresponds to 21	Corresponds to 30	Corresponds to 34
AFI (amniotic fluid index)	Normal	Normal	Normal
Placenta	High, third degree of readiness (marked calcinosis)	High on anterior wall	High on anterior wall
Fetal growth dynamics	Adequate	Adequate (23%)	Adequate (20%)
Duplex	Normal	Normal	Normal

## Discussion

Our presented patient highlights the complex medical management and ethical considerations in caring for a pregnant liver transplant recipient. Pregnancy in liver transplant recipients is considered high risk due to potential complications such as rejection, infection, and gestational hypertension [12]. Furthermore, pregnancy can affect the pharmacokinetics of immunosuppressive drugs, leading to inadequate immunosuppression or increased risk of liver failure [13].

In this case, the patient’s poor compliance with immunosuppressive therapy may have contributed to the poor liver function, leading to her hospitalization a year previously. Although her liver function blood tests were normal during the first trimester of pregnancy, her irregular use of immunosuppressive therapy may have contributed to the rapid deterioration of her liver function during the later trimester, leading to hepatic encephalopathy and the need for an urgent cesarean section. The premature birth of the baby was likely due to the mother’s poor liver function and liver failure. The patient’s irregular use of immunosuppressive therapy and failure to attend follow-up visits highlight the need for diligent adherence to treatment regimens and regular monitoring. The decision to perform an urgent caesarean section in this case highlights the ethical considerations in balancing maternal and fetal health. While the fetus was viable at 27/28 weeks, the mother’s rapidly deteriorating liver function and encephalopathy made the caesarean section a high-risk procedure. The decision to prioritize maternal health in this case was likely based on the understanding that the mother’s survival was necessary for the long-term well-being of the child.

The patient’s second pregnancy occurred 1 year after her first. At 14 weeks into the pregnancy, the patient was informed of possible complications by a multidisciplinary *concilium* consisting of a gastroenterologist, infectious disease specialist, gynecologist, and psychotherapist. Based on her previous history, the medical team was concerned about the potential for exacerbation of her condition during pregnancy. Additionally, the patient was taking medications including azathioprine and tacrolimus, which could potentially pose risks to the developing fetus. Despite their recommendation to terminate the pregnancy, the patient opted to continue. The patient’s decision to continue the pregnancy further complicated the medical management, as maintaining appropriate immunosuppression during pregnancy is crucial for maternal and fetal outcomes. The medical team then closely monitored the patient throughout her pregnancy, with a particular focus on managing her liver function and preventing potential complications.

The patient had an elective caesarean section at 37 weeks owing to a scar on uterus from a previous caesarean section and her preference. RDS prophylaxis was administered before the procedure, and a healthy baby girl was born. However, owing to blood loss during childbirth, the patient required two erythrocyte mass transfusions for anemia in the postpartum period.

Despite these challenges, on the fourth day after the caesarean section, the patient and child were discharged in a generally satisfactory condition. However, with close monitoring and management, the patient was able to successfully carry her pregnancy to term and deliver a healthy baby.

The case of the patient’s second pregnancy raises several important issues regarding reproductive healthcare and decision-making. The patient was informed of possible complications early in the pregnancy by a multidisciplinary *concilium* consisting of healthcare professionals from various specialties, but chose to continue with the pregnancy. This decision highlights the importance of informed consent and the autonomy of the patient in deciding what course of action to take with their health. Additionally, the patient had a complicated medical history, requiring ongoing medical treatment with multiple medications. The fact that the patient continued to take these medications during pregnancy and received methylprednisolone therapy while hospitalized raises questions about the potential risks and benefits of medication use during pregnancy and the importance of careful monitoring by healthcare professionals. The decision to perform a caesarean section at 37 weeks was made owing to a scar on the patient’s uterus from a previous caesarean section and her preference. This decision highlights the importance of individualized care and taking into account the patient’s medical history and preferences when making decisions about childbirth.

The postpartum period was also relatively complicated, with the patient requiring two erythrocyte mass transfusions for anemia. This highlights the potential risks and complications that can occur during and after childbirth, particularly in cases where there are underlying medical conditions or a complicated medical history.

Overall, the case of the patient’s second pregnancy highlights the importance of individualized care, informed consent, and careful monitoring by healthcare professionals in cases where there are potential complications or underlying medical conditions. It also highlights the potential risks and complications that can occur during and after childbirth, underscoring the importance of ongoing postpartum care and support for new mothers.

The management of pregnancy in women after liver transplantation requires a multidisciplinary team, including obstetricians, gastroenterologists, infectious disease specialists, and neonatologists [14]. The goals of management include maintaining maternal liver function and preventing complications such as preterm birth, preeclampsia, and fetal growth restriction, while also minimizing the risk of fetal exposure to immunosuppressive therapy [15].

The standard treatment protocol for a patient who has undergone a vascularized organ transplant typically involves a combination of a calcineurin inhibitor, mycophenolate, and a glucocorticosteroid.

When it comes to pregnancy, it is important to note that immunosuppressive therapy should not be stopped. To prevent organ rejection and potential loss, organ recipients must continue taking immunosuppressant medications throughout pregnancy [16, 17].

Several studies have evaluated the safety and efficacy of different immunosuppressive drugs during pregnancy in liver transplant recipients. However, these studies also noted the need for careful monitoring of drug levels during pregnancy and the potential for fetal complications, such as low birth weight and preterm birth.

Sivaprasadan *et al.* retrospectively reviewed all female liver transplant recipients who later conceived between 2006 and 2019. Of the 750 liver transplantations performed, 129 were female, and 62 of them were in the reproductive age group. A total of seven pregnancies occurred during the study period. All patients were on tacrolimus monotherapy, and none of them had rejection during pregnancy despite a low median tacrolimus trough level of 2.7 ng/mL. Live births occurred in six of the seven pregnancies, with a median gestational age of 37.5 weeks. The study also found that there is a physiological reduction of tacrolimus trough levels during pregnancy, for which dose augmentation is not usually required [18].

In addition, Jabiry-Zieniewicz *et al.* conducted a retrospective study of liver transplant recipients who became pregnant between 1992 and 2014 at a single medical center in Poland. The study included 24 pregnancies in 20 women, and the authors evaluated maternal and fetal outcomes, as well as the impact of immunosuppressive therapy on pregnancy. The study found that the majority of pregnancies resulted in live births, and that maternal and fetal outcomes were generally good. However, there were some cases of preterm delivery, low birth weight, and fetal growth restriction. The authors also noted that the use of immunosuppressive therapy during pregnancy was associated with some risks, including increased rates of gestational diabetes and preeclampsia [19].

Rinella further discussed the need for close monitoring of immunosuppressant drug levels during pregnancy, as the pharmacokinetics of these drugs can be altered owing to changes in metabolism and renal function. The author recommends minimizing the use of mycophenolate mofetil and sirolimus during pregnancy owing to their teratogenic effects. Tacrolimus and cyclosporine are preferred immunosuppressive agents during pregnancy, as they have been associated with good maternal and fetal outcomes [20].

In addition to appropriate immunosuppressive therapy, careful monitoring of maternal and fetal health is crucial in pregnant liver transplant recipients. Regular ultrasound monitoring can help detect potential complications such as gestational hypertension, placental insufficiency, and fetal growth restriction [4]. In this case, the patient’s delayed follow-up and subsequent admission to the hospital with abdominal pain and jaundice likely indicate a failure in monitoring maternal health.

## Conclusion

Pregnancy in women after liver transplantation requires close monitoring of maternal liver function, immunosuppressive therapy, and fetal growth. The multidisciplinary management of such pregnancies is necessary to prevent adverse outcomes. The case presented highlights the importance of patient compliance with immunosuppressive therapy during pregnancy and the potential consequences of poor compliance. This case report also underscores the importance of regular follow-up visits, strict adherence to immunosuppressive therapy, and vigilant monitoring in graft patients. It also emphasizes the need for a multidisciplinary approach and thorough prenatal counseling in managing high-risk pregnancies in this population. A personalized approach is necessary to balance the potential benefits of childbirth with the risks involved in pregnancy after liver transplantation. Further research and clinical experience will continue to provide valuable insights into this important area of medical practice.

## Data Availability

The authors confirm that the raw data and other additional information are available from the corresponding author upon request.
